# SVM-based prediction of linear B-cell epitopes using Bayes Feature Extraction

**DOI:** 10.1186/1471-2164-11-S4-S21

**Published:** 2010-12-02

**Authors:** Lawrence JK Wee, Diane Simarmata, Yiu-Wing Kam, Lisa FP Ng, Joo Chuan Tong

**Affiliations:** 1 Singapore Immunology Network, 8A Biomedical Grove, #04-06 Immunos, Biopolis, Singapore 138648; 2 Data Mining Department, Institute for Infocomm Research, 1 Fusionopolis Way, #21-01 Connexis South Tower, Singapore 138632; 3 Department of Biochemistry, Yong Loo Lin School of Medicine, National University of Singapore, 8 Medical Drive, Singapore 117597

## Abstract

**Backgound:**

The identification of B-cell epitopes on antigens has been a subject of intense research as the knowledge of these markers has great implications for the development of peptide-based diagnostics, therapeutics and vaccines. As experimental approaches are often laborious and time consuming, *in silico* methods for prediction of these immunogenic regions are critical. Such efforts, however, have been significantly hindered by high variability in the length and composition of the epitope sequences, making naïve modeling methods difficult to apply.

**Results:**

We analyzed two benchmark datasets and found that linear B-cell epitopes possess distinctive residue conservation and position-specific residue propensities which could be exploited for epitope discrimination *in silico*. We developed a support vector machines (SVM) prediction model employing Bayes Feature Extraction to predict linear B-cell epitopes of diverse lengths (12- to 20-mers). The best SVM classifier achieved an accuracy of 74.50% and A_ROC_ of 0.84 on an independent test set and was shown to outperform existing linear B-cell epitope prediction algorithms. In addition, we applied our model to a dataset of antigenic proteins with experimentally-verified epitopes and found it to be generally effective for discriminating the epitopes from non-epitopes.

**Conclusion:**

We developed a SVM prediction model utilizing Bayes Feature Extraction and showed that it was effective in discriminating epitopes from non-epitopes in benchmark datasets and annotated antigenic proteins. A web server for predicting linear B-cell epitopes was developed and is available, together with supplementary materials, at http://www.immunopred.org/bayesb/index.html.

## Background

Humoral immune responses play critical roles in the body’s defense against pathogens and foreign agents, as well as certain hypersensitivity reactions [1]. The principal agents of the humoral immune responses are the B lymphocytes (B-cells). Naïve B-cells are stimulated by specific recognition and binding of the B-cell receptor to a region on the antigen called the epitope. Together with co-stimulation from the T lymphocytes (T-cells), naïve B-cells become fully activated and go on to proliferate and differentiate into memory and plasma cells, with the latter serving as the key engines for producing specific antibodies. The identification and mapping of B-cell epitopes on antigens has been a subject of intense research as the knowledge of these markers has profound implications for the development of peptide-based diagnostics, therapeutics and vaccines. B-cell epitopes may comprise of linear, contiguous stretches of amino acids within a protein, or they can be discontinuous stretches of amino acids that are brought together spatially by protein folding. Although the majority of B-cell epitopes are discontinuous in nature, difficulties in the design of such epitopes have led to an emphasis on the identification of linear B-cell epitopes. As experimental efforts for linear B-cell epitope identification is often laborious and expensive, much effort has been devoted to develop *in silico* models for epitope prediction.

The pioneering methods for linear B-cell epitope prediction were based on correlations between several physicochemical properties of amino acids and the locations of the epitopes on antigens. Computational methods such as PREDITOP [2], PEOPLE [3], BEPITOPE [4] and BcePred [5] implemented a variety of physicochemical propensity scales, such as hydrophilicity, flexibility or solvent accessibility, in their prediction models. However, Blythe and Flower [6] conducted an extensive investigation of the utility of these physicochemical propensity scales for predicting linear B-cell epitopes and concluded that even the best set of scales and parameters performed only marginally better than random and the reported performance of these methods were likely to be overly optimistic. Interestingly, several methods based on machine learning algorithms were explored thereafter and were shown to improve prediction performance over the earlier methods: Larson *et al.*[7] developed BepiPred which uses two amino acid propensity scales and hidden markov models (HMM); Sollner and Mayer [8] utilized decision trees and the nearest-neighbor method; and Saha and Raghava [9] experimented with artificial neural networks. More recently, various groups have shown improved prediction performance with the use of the support vector machines (SVM) algorithm. Chen *et al.*[10] found that certain amino acid pairs were found to occur more frequently in B-cell epitopes and developed a SVM method based on amino acid pairs propensities, achieving the best accuracy value of 71%. EL-Manzalawy *et al.*[11] reported superior performance over previous methods when they utilized string kernels in the SVM algorithm, achieving the highest A_ROC_ (area under the receiver operating characteristic curve) score of 0.758. COBEpro utilized unique feature representations of epitope sequences in the SVM algorithm and realized an A_ROC_ of 0.829 [12]. In addition, Rubinstein *et al.*[13,14] developed a comparatively accurate model for predicting immunogenic regions on a protein’s three dimensional structure or sequence using the Naïve Bayes classifier.

In this paper, B-cell epitopes and non-epitopes from benchmark datasets were analyzed to identify unique sequence characteristics which can be exploited for epitope discrimination. We observed that specific amino acids were unusually enriched or depleted in epitopes compared to non-epitopes. Moreover, epitopes were found to possess unique patterns of position-specific residue propensities. We developed a SVM prediction model utilizing Bayes Feature Extraction and predicted linear B-cell epitopes of diverse lengths (12- to 20-mers). The model was found to achieve superior results when compared to other existing methods. The best SVM classifier achieved an accuracy of 74.50% (sensitivity of 81%, specificity of 68%) and A_ROC_ of 0.84 when tested on an independent dataset. In addition, the model was tested on dataset of experimentally verified antigens with annotated epitopes and was found to be generally effective in discriminating the epitopes from non-epitopes. The prediction model is implemented on a web server, BayesB, which can be freely accessed at http://www.immunopred.org/bayesb/index.html.

## Results and Discussion

### Sequence analysis of linear B-cell epitopes

Accurate *in silico* prediction of linear B-cell epitopes is a highly desirable goal as it is often the important first step in computational mapping of epitopes on pathogen proteins and in the design of immuno-diagnostics and vaccine development. Much of the existing work has been centered on the use of raw sequence information as well as the innate physicochemical properties of the sequences as represented by a multitude of amino acid propensity scales. While Blythe and Flower have shown that the use of such propensity scales by themselves offer limited predictive capabilities and do not significantly improve prediction accuracy, recent methods involving machine learning algorithms suggest that incorporating such information into more complex, non-linear models might be helpful. Interestingly, while different feature representations were applied to predict linear B-cell epitopes, there are only limited insights on the use of residue conservation and position-specific amino acid profiles for prediction. As these features were found to be useful for improving prediction accuracy in several related biological domains [15-17], it is intuitive to question if these features are similarly prominent in linear B-cell epitopes and could be exploited to enhance the performance of machine learning-based prediction models.

We conducted a series of analyses on epitope sequences using benchmark datasets from EL-Manzalawy *et al.*[11] and Chen *et al.*[10] (henceforth termed as EL-Manzalawy and Chen datasets respectively). Both datasets contain linear B-cell epitope sequences derived from Bcipep [18]. From both datasets, we extracted 20-mer epitope and non-epitope peptides for our analyses. Our choice of 20-mer peptides is based on the fact that most epitopes (~86%) in both datasets are 20 amino acids or shorter in length, with 20-mer peptides being found in the greatest proportion. A sizeable number of shorter epitopes are also enclosed within the longer 20-mer peptides. In addition, 20-mer peptides were widely used in earlier algorithmic development so the usage of these peptides would be convenient for comparative analyses [10,11].

**Figure 1:**
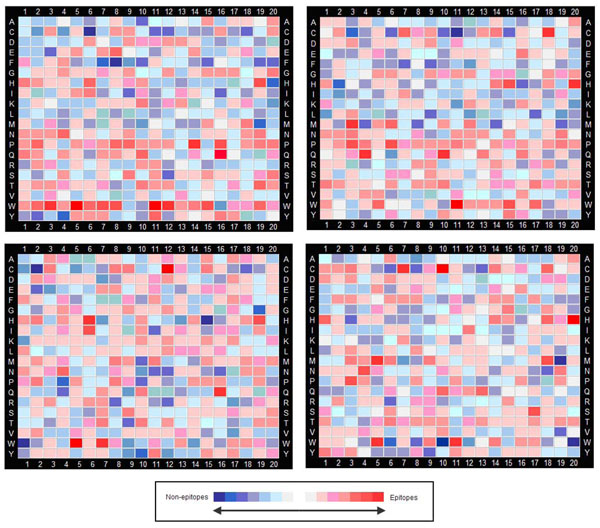
**Heat maps of relative position-specific amino acid propensities (P_x_).** P_x_ values of amino acids were computed for EL-Manzalawy analysis and control subsets (left, top and bottom respectively) and Chen analysis and control subsets (right, top and bottom respectively). P_x_ values were computed as the ratio of the frequency of occurrence of the amino acid in the epitopes pool over the frequency of occurrence of the same amino acid in the non-epitopes pool at a specific position. Px values were calculated using the epitopes and non-epitope pools in the analysis subsets, and using the two pools of non-epitopes in the control subsets. Increasing color intensities in the red spectrum indicate enrichment in epitopes pool (high P_x_) while increasing color intensities in the blue spectrum indicate enrichment in non-epitopes pool (low P_x_).

Next, we constructed an analysis subset containing a pool of 20-mer epitopes and an equivalent-sized pool of 20-mer non-epitopes from the EL-Manzalawy dataset. An analysis subset was also constructed from Chen dataset in the same manner. To further validate the analyses, we constructed two control subsets from EL-Manzalawy and Chen datasets respectively. The control subsets contain only non-epitopes data which is divided into two pools of equal numbers each. For both analysis and control subsets, we calculated the relative position-specific propensities of each amino acid (P_x_) at twenty residue positions on the 20-mer peptides. For the analysis subsets, P_x_ indicates the relative frequency of a particular amino acid in the epitopes pool over the frequency of the same amino acid in the non-epitopes pool at the same position. For the control subsets, P_x_ values were calculated in the same manner but using the two pools of non-epitopes. P_x_ values were plotted as heat maps to facilitate visualization (Figure 1). For the EL-Manzalawy dataset (Figure 1, left top and bottom), several amino acids were found to have very distinctive P_x_ scores in the analysis subset compared to the control subset. Across the entire length of the 20-mer peptides, tryptophan (W), proline (P), glutamine (N) were found to be relatively enriched in epitopes, with the average P_x_ of 2.16, 1.59 and 1.24 respectively (Table of average P_x_ scores for amino acids across whole peptide length is provided in Additional file 1). On the other hand, phenylalanine (F) and leucine (L) were found to be relatively depleted in epitopes with the average P_x_ of 0.77 and 0.80 respectively. Similarly, for the Chen dataset (Figure 1, right top and bottom), W, P and N were found to be relatively enriched in epitopes with the average P_x_ of 1.50, 1.57 and 1.22 respectively while F and L residues were observed to be relatively depleted in epitopes with average P_x_ of 0.77 for both.

**Figure 2:**
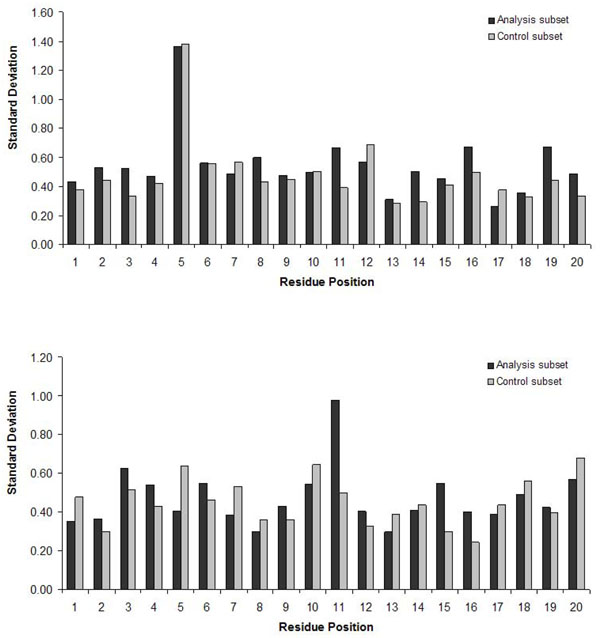
**Quantitative measurement of the spread between the relative position-specific amino acid propensities (P_x_) at various residue positions in analysis and control subsets.** At each residue position, standard deviations of P_x_ of all amino acids were calculated. Standard deviation scores were plotted against analysis and control subsets for EL-Manzalawy (*top*) and Chen (*bottom*) datasets.

As certain amino acids are distinctly conserved in epitopes, we further questioned if there are patterns of position-specific conservation of residues that are unique among the epitopes. To this end, we compared the standard deviations of P_x_ of all amino acids at each position along the 20-mer peptides in the analysis and control subsets. It could be reasoned that higher variability of relative position-specific propensities of amino acids in epitopes is indicative of distinct and perhaps complex patterns of position-specific conservation of residues. Indeed, for the EL-Manzalawy dataset (Figure 2, top), the standard deviations of P_x_ were notably greater for 75% (15/20) of the residue positions for peptides in the analysis subset compared to the control subset. For the Chen dataset (Figure 2, bottom), 50% (10/20) of all residue positions were found to have greater standard deviations of P_x_ in the analysis subset compared to the control subset. These results indicate that there might be complex patterns of position-specific residue propensities in epitope sequences which could be exploited further to enhance computational prediction.

### SVM-based prediction with Bayes Feature Extraction

To encapsulate these complex patterns of residue conservation and position-specific propensities for computational prediction, we constructed SVM prediction models using features extracted using the Bayes Feature Extraction (BFE) approach recently introduced by Shao *et al.*[19]. BFE was shown to significantly improve prediction of protein methylation sites [19] and caspase cleavage sites [20] over using simple binary encoding schemes. Essentially, in the application of BFE, feature vectors are encoded in a bi-profile manner containing attributes from positive position-specific and negative position-specific profiles. These profiles are generated through calculating the frequency of occurrence of each amino acid at each position of the peptide sequence in the epitopes pool and non-epitopes pool respectively. We tested our SVM prediction model using the EL-Manzalawy dataset which is suggested to be more appropriately suited for benchmarking purposes as it is comprised of homology-reduced epitope sequences. We constructed SVM classifiers for peptides of diverse lengths from the EL-Manzalawy dataset (12- to 20-mers) using simple binary encoding or BFE schemes (detailed in the Materials and Methods). Sequences were partitioned respectively into training and independent test sets. 10-fold cross-validation was conducted on training sets to obtain the optimal set of SVM parameters (results and choice of optimal parameters are detailed in Additional file 2). The SVM classifiers are trained on the entire training set using the optimized parameters and evaluated for their performances on the independent test sets. As shown in Table 1, simple binary encoding schemes were shown to achieve mediocre performances as accuracy and A_ROC_ scores of all peptide lengths (represented as SVM12, SVM14, SVM16, SVM18 and SVM20 respectively) were consistently below 60% and 0.70 respectively. However, when simple binary encoding schemes were replaced with BFE (represented as BFE-SVM12, BFE-SVM14, BFE-SVM16, BFE-SVM18 and BFE-SVM20 respectively), significant improvements were attained across all peptide lengths. In these cases, accuracy and AROC scores for each peptide length were consistently higher than those obtained from classifiers trained using simple binary encoding schemes, with the best classifier (BFE-SVM20) achieving an accuracy of 74.50%, sensitivity of 81%, specificity of 68% and A_ROC_ of 0.84

**Table 1:** Results of SVM prediction on independent test sets

SVM Classifier*	Sensitivity (%)	Specificity (%)	Accuracy (%)	A_ROC_
SVM12	59.00	54.00	56.50	0.60
SVM14	60.00	51.00	55.50	0.62
SVM16	62.00	47.00	54.50	0.59
SVM18	62.00	57.00	59.50	0.67
SVM20	54.00	62.00	58.00	0.64
BFE-SVM12	70.00	64.00	67.00	0.71
BFE-SVM14	63.00	69.00	66.00	0.73
BFE-SVM16	73.00	62.00	67.50	0.73
BFE-SVM18	70.00	69.00	69.50	0.80
BFE-SVM20	81.00	68.00	74.50	0.84
BFE-Chen	70.00	67.00	68.50	0.74

*SVM classifiers are generated for different peptide lengths as indicated by the numerical value assigned to each classifier.

Next, we compared our SVM prediction model with algorithms developed by EL-Manzalawy *et al.*[11], Sweredoski and Baldi [12] and Chen *et al.*[10], as they are among the best performing algorithms in this domain. Since the EL-Manzalawy dataset was utilized in some of these algorithms, it was relatively convenient to perform benchmark comparisons with our model. In EL-Manzalawy *et al.*[11], the SVM classifier trained on the 20-mer peptide dataset using string kernels was found to achieve overall accuracy of 67.90% and A_ROC_ of 0.758 on five-fold cross-validation. For classifiers trained on the 12- to 18-mer datasets, A_ROC_ scores ranged from 0.709 to 0.751. Interestingly, in our prediction model, A_ROC_ scores were similarly observed to improve generally as the length of the peptide increases for both representation schemes. It was suggested that many of the shorter epitopes are part of the longer epitopes and the presence of additional amino acids flanking the shorter epitopes might assist the classifier in discriminating the epitopes from non-epitopes [11]. In Sweredoski and Baldi [12], the SVM classifier trained on the same EL-Manzalawy dataset using a proprietary feature extraction scheme achieved accuracy and A_ROC_ of 71.4% and 0.768 respectively on five-fold cross-validation. Chen *et al.*[10] reported accuracy of 71.0% on five-fold cross-validation using amino acid pair antigenicity propensities and SVM. To benchmark against Chen *et al.*[10], we tested our model using the Chen dataset (20-mers) and achieved accuracy and A_ROC_ scores of 68.50% and 0.74 respectively (Table 1, BFE-Chen). While it appears that our SVM implementation using Chen dataset performed slightly poorer when compared to the corresponding test on EL-Manzalawy dataset (BFE-SVM20) and with Chen *et al.*[10], we reasoned that this was due to the relatively higher proportion of similar sequences in the Chen dataset compared to the EL-Manzalawy dataset.

To further validate the ability of our SVM model to discriminate epitopes from non-epitopes, we applied the BFE-SVM20 classifier to predict linear B-cell epitopes on 14 antigen sequences derived from a dataset curated by Pellequer *et al.*[2,7] (henceforth termed as Pellequer dataset). We have chosen to apply the BFE-SVM20 classifier as it was shown to achieve the best performance in our earlier validation. Antigen sequences were scanned using a 20-mer sliding window and each 20-mer peptide was predicted as an epitope or not. The prediction outputs were integrated and re-computed as a residue epitope propensity score, *E_p_* for each residue on the antigen where residues with *E_p_* > 0 were assigned as epitopes and *E_p_* ≤ 0 were assigned as non-epitopes. In this representation, every residue on the antigen sequence is scored for its propensity to be an epitope or non-epitope. This facilitates the identification of epitopes of different lengths on antigen sequences as well as regions which might be epitope-rich. As shown in Table 2, the use of residue epitope propensity scores was able to achieve above 50% accuracy for most of the antigens (11/14) and above 70% accuracy for four antigens. In addition, from our analysis of the residue epitope propensity scores on all antigens (data not shown), we noted that experimentally annotated epitopes had generally higher *E_p_* scores compared to non-epitopes (1.53 versus -0.23 respectively, *P-value* <0.1).

**Table 2:** SVM prediction of epitopes on antigens in Pellequer dataset

UniProt ID	Antigen	Accuracy (%)
P01556	Enterotoxin beta chain precursor (Cholera)	76.74
P00001	Cytochrome c (Human)	53.03
P03138	Major surface antigen precursor (Hepatitis B virus)	37.32
P01233	Choriogonadotropin beta chain precursor (Human)	44.09
P01574	Interferon beta precursor (Human)	72.48
P02238	Leghemoglobin A (Soybean)	75.24
P00698	Lysozyme C precursor (Chicken)	66.06
P02247	Myohemerythrin (Themiste zostericola)	61.25
P02185	Myoglobin (Physeter catodon)	66.96
P04127	PAP fimbrial major pilin protein precursor (E.coli)	58.50
P01112	Transforming protein p21/H-RAS-1 (Human)	83.44
P00797	Renin precursor (Human)	36.68
P01484	Neurotoxin II (Androctonus australis hector)	51.06
P03570	Coat protein (Tobacco mosaic virus)	52.50

In summary, we showed that SVM-based prediction using Bayes Feature Extraction is an effective method for predicting linear B-cell epitopes. By using a set of benchmark datasets, this method was observed to perform better than several of the best existing methods. Also, by scoring experimentally-verified antigen sequences with residue epitope propensity scores derived from the SVM prediction outputs, annotated epitope and non-epitope regions on the antigens could be discriminated effectively. The application of Bayes Feature Extraction consistently enhanced SVM prediction across various peptide lengths, suggesting that more informative representation of conserved sequence characteristics is facilitated by this approach. In Bayes Feature Extraction, the encoding of bi-profile feature vectors assumes that information carried by each residue is independent of the other residues. Interestingly, in linear B-cell epitopes, specific di-peptides were found to be enriched [10], suggesting that the type of amino acid found at a particular residue position on the epitope may somewhat be correlated to type of amino acid found at the adjacent positions. Therefore, it is tempting to speculate if prediction performance can be improved if amino acid di-peptides information is factorized together with Bayes features in the SVM classifier. These findings also corroborate previous studies on the use of feature selection methods to improve prediction performance [15-17]. Interestingly, feature extraction using the Bayes Feature Extraction approach as implemented here is intuitively similar to the application of position-specific scoring matrix (PSSM) profiles for predicting protective linear B-cell epitopes by EL-Manzalawy *et al.*[21]. In that study, it was shown that the Naive Bayes classifier trained using PSSM profiles significantly outperformed the propensity scale-based methods and simple binary encoding with SVM in predicting protective linear B-cell epitopes. Separately, Song and co-workers observed improved performance when PSI-BLAST profiles were used as input features to train SVM classifiers for predicting residue depth and cis/trans isomerization in proteins [16,17]. It is expected that future work on feature selection and feature extraction methods would be helpful here and in related bioinformatics domains.

## Conclusion

In this paper, we analyzed benchmark datasets and report that linear B-cell epitopes and non-epitope sequences have distinctive residue composition and position-specific propensity patterns which could be used for epitope discrimination *in silico*. We developed a SVM prediction model employing Bayes Feature Extraction to predict linear B-cell epitopes of diverse lengths. The best SVM classifier achieved an accuracy of 74.50% and A_ROC_ of 0.84 on an independent test set and was shown to perform better than several existing linear B-cell prediction algorithms. In addition, we applied our method on a dataset of antigenic proteins with experimentally-verified epitopes and found it to be generally effective for discriminating the epitopes from non-epitopes. To complement experimental research, we have implemented our prediction model on a web server, BayesB, which is freely accessible at http://www.immunopred.org/bayesb/index.html.

As publicly available experimental data on B-cell epitopes accumulates, we plan to periodically re-train and re-evaluate our SVM prediction model. We have also begun comparative studies where B-cell epitope predictions of clinically important viral proteomes are cross-validated with the antigenicity potential of actual proteome-derived peptides. It is expected that these studies would provide a richer evaluation of the real-world performance of our SVM prediction model.

## Materials and Methods

### Datasets

We obtained two benchmark datasets of linear B-cell epitopes and non-epitopes from EL-Manzalawy *et al.*[11] (henceforth termed as the EL-Manzalawy dataset) and Chen *et al.*[10] (Chen dataset). EL-Manzalawy dataset contained 701 unique, homology-reduced epitopes of five different peptide lengths (12-, 14-, 16-, 18- and 20-mers) and Chen dataset contained 872 unique 20-mer epitopes. In both datasets, equal numbers of non-epitope sequences were deposited by the authors through randomly extracting peptides from sequences in Uniprot databases [22] while ensuring that none of them were included among the epitopes.

We constructed an analysis subset containing a pool of 20-mer epitopes and an equivalent-sized pool of 20-mer non-epitopes from EL-Manzalawy dataset. An analysis subset was also constructed from Chen dataset in the same manner. To further validate the analyses, we constructed two control subsets from EL-Manzalawy and Chen datasets respectively. The control subsets contain only non-epitopes data which is divided into two pools of equal numbers each. Therefore, for the EL-Manzalawy dataset, the analysis subset comprised of a pool of 351 epitope and a pool of 350 non-epitope sequences, while the control subset contained two pools of non-epitopes with 350 sequences in each. For the Chen dataset, the analysis subset comprised of a pool of 436 epitope and a pool of 436 non-epitope sequences while the control subset contained two pools of 436 non-epitopes in each.

For SVM training and testing, peptides from EL-Manzalawy dataset (12- to 20-mers) were divided into training and independent test sets which comprised of 601 epitopes/601 non-epitopes and 100 epitopes/100 non-epitopes respectively. Peptides from Chen dataset (20-mers) were divided into training and independent test sets which comprised of 736 epitopes/736 non-epitopes and 100 epitopes/100 non-epitopes respectively.

### Relative position-specific amino acid propensities

The relative position-specific amino acid propensity, P_x_, of an amino acid is a quantitative indicator of the propensity of the amino acid to be found at a particular position on the epitope. It is defined as the ratio of the frequency of occurrence of the amino acid in the epitopes pool to the frequency of occurrence of the same amino in the non-epitopes pool at a specific position. As 20-mer peptides were used, P_x_ values were calculated for every amino acid at each of the twenty residue positions and visualized on heat maps. P_x_ values were calculated using the epitopes and non-epitope pools in the analysis subsets, and using the two pools of non-epitopes in the control subsets. Average P_x_ of a specific amino acid is calculated by taking the average of all P_x_ values of that amino acid across all positions on the 20-mer peptides.

### Vector encoding schemes

To encapsulate sequence information into a format for SVM training and testing, the sequences were coded as input vectors in simple binary format or in a bi-profile manner using Bayes Feature Extraction. In simple binary encoding, each amino acid is represented by a 20-dimensional vector, composed of either zero or one as elements. For example, alanine was represented as [0,0,0,0,0,0,0,0,0,0,0,0,0,0,0,0,0,0,0,1] and cysteine as [0,0,0,0,0,0,0,0,0,0,0,0,0,0,0,0,0,0,1,0]. Therefore, in this case, a 20-mer peptide will encoded by a 400-dimensional vector (20 x 20). For details on bi-profile vector encoding using Bayes Feature Extraction, readers are advised to refer to Shao *et al.*[19]. Briefly, feature vectors are encoded in a bi-profile manner containing attributes from positive position-specific and negative position-specific profiles. These profiles are generated through calculating the frequency of occurrence of each amino acid at each position of the peptide sequence in the epitopes pool and non-epitopes pool respectively. Therefore, a 20-mer input peptide will be encoded by a 40-dimensional (20 x 2) feature vector containing information on the residues of the peptide in the positive (epitope) and negative (non-epitope) spaces.

### SVM Training and Testing

For SVM implementation, we used the freely downloadable LIBSVM package by Chang and Lin [23]. Details of the SVM methodology can be obtained from the article by Burges [24]. In short, SVM is based on the structural risk minimization principle from statistical learning theory. A set of positive and negative examples can be represented by the feature vectors *x_i_* (*i* = 1, 2,....N) with corresponding labels *y_i_* ∈ {+1,-1}. To classify the data, the SVM trains a classifier by mapping the input samples, using a kernel function in most cases, onto a high-dimensional space, and then seeking a separating hyperplane that differentiates the two classes with maximal margin and minimal error. The decision function for new predictions on unseen examples is given as:

where K (*x_i_*·*x_j_* ) is the kernel function, and the parameters are determined by maximizing the following:

under the conditions,

The variable C serves as the regularization parameter that controls the trade-off between margin and classification error. We used the radial basis function (RBF) kernel and performed parameter optimization for γ, which determines the capacity of the RBF kernel, and the regularization parameter C using 10-fold cross-validation on EL-Manzalawy and Chen training sets (optimization process is further described in Additional file 2). In 10-fold cross-validation, the training dataset was spilt into 10 subsets where one of the subsets was used as the test set while the other subsets were used for training the classifier. The trained classifier was tested using the test set. The process is repeated ten times using a different subset for testing, hence ensuring that all subsets are used for both training and testing. The optimal values of γ and C obtained from the optimization processes were used subsequently for training the entire training sets to create the final SVM classifier for testing on the independent test sets.

### Performance metrics

Various quantitative variables were employed to measure the effectiveness of the SVM model for predicting linear B-cell epitopes:

(i) TP, true positives - the number of correctly classified epitopes.

(ii) FP, false positives - the number of incorrectly classified non-epitopes.

(iii) TN, true negatives - the number of correctly classified non-epitopes.

(iv) FN, false negatives - the number of incorrectly classified epitopes.

Using the variables above, the metrics Sensitivity (*S_n_*) and Specificity (*S_p_*)*,* which indicate the ability of the prediction model to correctly classify the epitope and non-epitope sequences respectively, were computed:

To provide an indication of the overall performance of the prediction model, we computed Accuracy (*A_cc_*):

While these metrics are generally indicative of model performance, they are dependent on the decision threshold. Therefore, a threshold-independent metric, the area under the receiver operating characteristic curve (A_ROC_) was computed as well.

### Residue epitope propensities and performance evaluation on Pellequer dataset

We define ‘residue epitope propensity’ or *E_p_,* as a quantitative measure of the likelihood of a residue to be part of an epitope. *E_p_* scores are computed to evaluate the effectiveness of the prediction model on antigens with known epitopes. 14 antigens with experimentally verified epitopes from a dataset derived from Pellequer *et al.*[2,7] were scanned by a 20-mer sliding window and predicted for linear B-cell epitopes using the BFE-SVM20 classifier. SVM output scores from each 20-mer sliding window were integrated and re-computed as the residue epitope propensity score, *E_p_* for each residue on the antigen. Here, *E_p_* of a residue is computed as the additive sum of the SVM output scores from the prediction of the 20-mer sliding windows with the residue located at different positions along the sliding window. The *E_p_* values were calculated for residues in all antigen sequences from the 20^th^ residue up the last 20^th^ residue. To measure its effectiveness as a predictive metric, *E_p_* was benchmarked against the annotated epitopes on the antigens. Residues with *E_p_ >* 0 and annotated as epitope were assigned as true positives (*TP*) while residues with *E_p_ ≤* 0 and not annotated as epitope were assigned as true negatives (*TN*). Residues with *E_p_ ≤ 0* and annotated as epitope were assigned as false negatives (*FN*) while residues with *E_p_ >* 0 and annotated as non-epitope were assigned as false positives (*FP*)*.* To measure prediction performance, accuracy scores were computed as described earlier for each antigen using the *TN*, *TN*, *FN* and *FP* variables.

## Competing Interests

The authors declare that they have no competing interests.

## Authors’ Contributions

LJKW conceived the idea for utilizing Bayes Feature Extraction for SVM prediction of linear B-cell epitopes. JCT, LFPN, DS, YWK contributed with ideas on the experimentation and assisted with the drafting of the manuscript. All authors read and approved the final manuscript.

## Supplementary Material

Additional file 1 Description of Data: Average P_x_ of each amino acid is calculated by averaging the P_x_ values of the particular amino acid across all residue positions on the 20-mer peptides from analysis and control subsets of EL-Manzalawy and Chen datasets. Click here for file

Additional file 2 Description of Data: Training sets from EL-Manzalawy and Chen datasets were trained under 10-fold cross-validation using various C and γ values. Optimal set of C and γ values for each peptide representation is indicated below the plot. Click here for file
