# Supplementary material for: SVM-based prediction of linear B-cell epitopes using Bayes Feature Extraction
Source: BMC Genomics. 2010 Dec 2;11(Suppl 4):S21. doi: 10.1186/1471-2164-11-S4-S21 (PMC3005920; doi:10.1186/1471-2164-11-S4-S21)
Supplement: Additional file 1 — Description of Data: Average Px of each amino acid is calculated by averaging the Px values of the particular amino acid across all residue positions on the 20-mer peptides from analysis and control subsets of EL-Manzalawy and Chen datasets. [file 1471-2164-11-S4-S21-S1.pdf]

Average P_x of amino acids: Average P_x of each amino acid is calculated by averaging the P_x values of the particular amino acid across all residue positions on the 20-mer peptides from analysis and control subsets of EL-Manzalawy and Chen datasets.

	Average P_x			
	EL-Manzalawy		Chen	
**Amino acid**	**Analysis**	**Control**	**Analysis**	**Control**
A	0.95	1.07	0.92	0.93
C	0.87	1.11	1.13	1.39
D	1.04	1.07	1.03	1.07
E	1.07	0.99	1.00	1.00
F	0.77	1.02	0.77	1.11
G	1.21	1.05	1.21	0.99
H	1.14	1.19	1.11	1.26
I	0.95	1.12	0.85	1.00
K	1.01	1.13	0.96	0.92
L	0.80	1.08	0.77	1.02
M	0.93	1.20	1.10	1.25
N	1.24	0.98	1.22	1.10
P	1.60	1.10	1.57	1.08
Q	1.27	1.04	1.27	1.10
R	0.93	0.96	1.03	0.99
S	1.13	1.08	1.05	1.13
T	1.25	1.08	1.35	1.27
V	0.96	0.98	0.99	1.03
W	2.16	1.38	1.50	1.15
Y	1.09	0.99	1.01	1.06
