# Supplementary material for: SVM-based prediction of linear B-cell epitopes using Bayes Feature Extraction
Source: BMC Genomics. 2010 Dec 2;11(Suppl 4):S21. doi: 10.1186/1471-2164-11-S4-S21 (PMC3005920; doi:10.1186/1471-2164-11-S4-S21)
Supplement: Additional file 2 — Description of Data: Training sets from EL-Manzalawy and Chen datasets were trained under 10-fold cross-validation using various C and γ values. Optimal set of C and γ values for each peptide representation is indicated below the plot. [file 1471-2164-11-S4-S21-S2.pdf]

SVM parameter optimization: Training sets from EL-Manzalawy and Chen datasets were trained under 10-fold cross-validation using various C and γ values. Optimal set of C and γ values for each peptide representation is indicated below the plot.

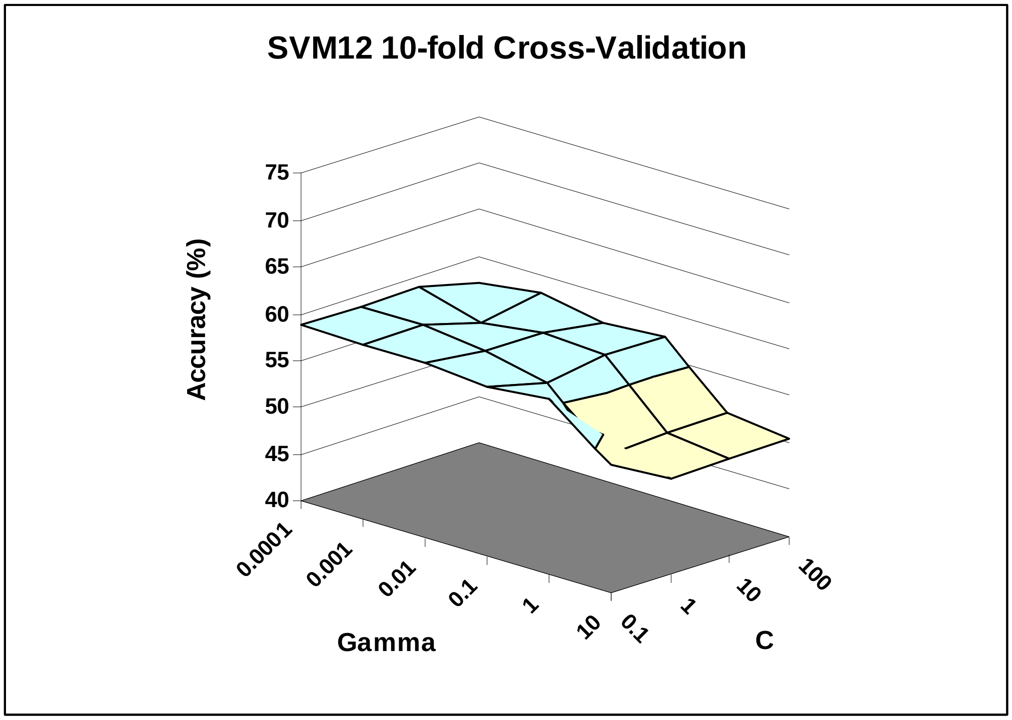

C=0.1 and γ =0.0001

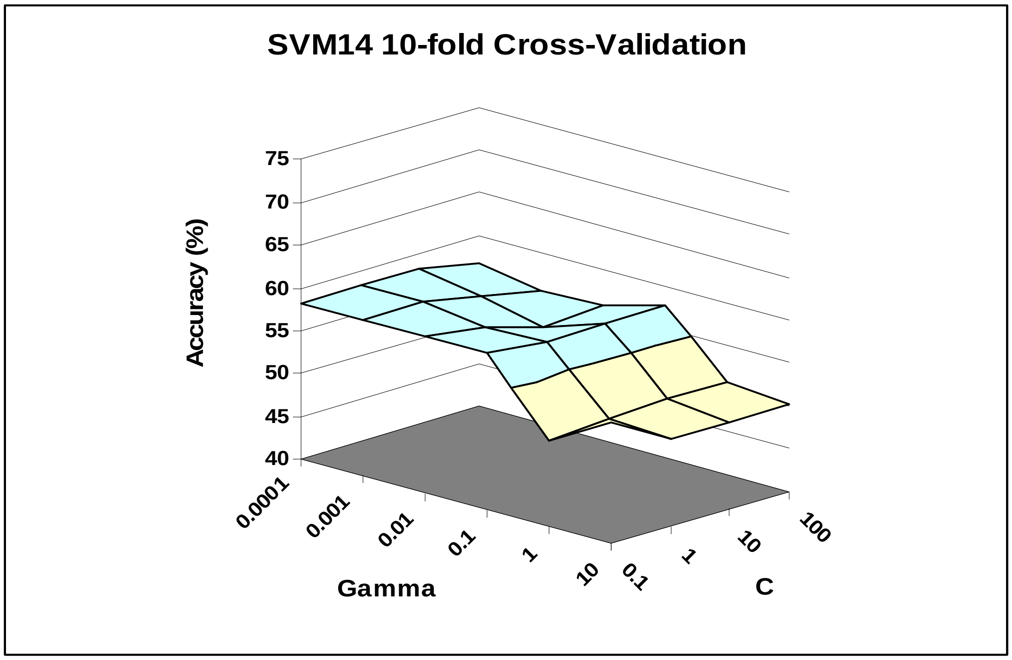

C=0.1 and γ =0.0001

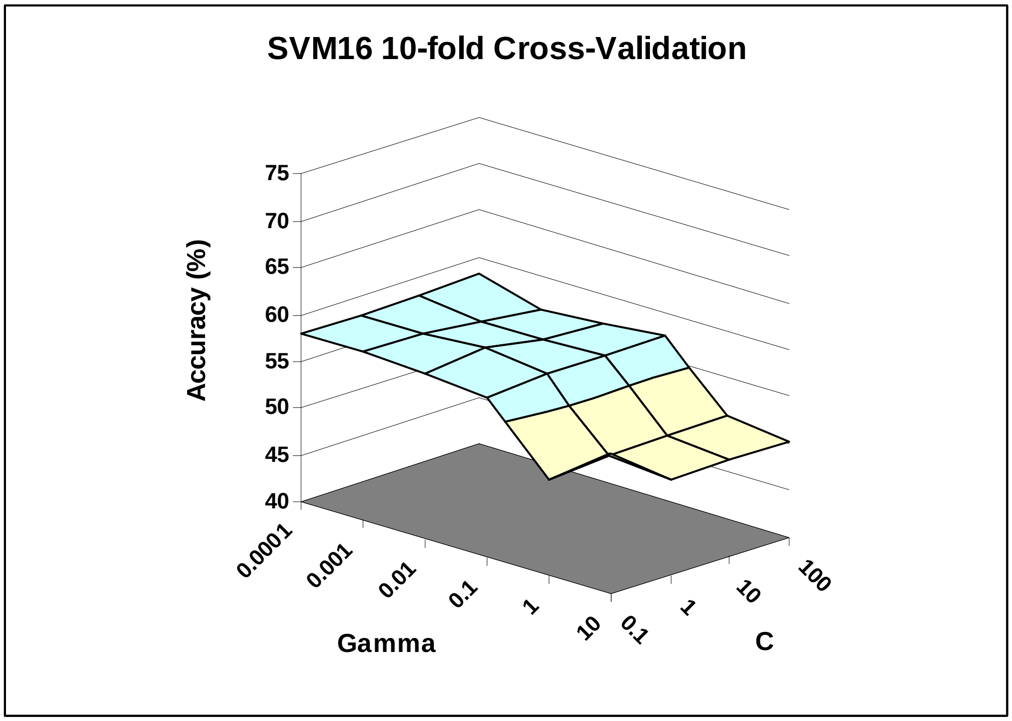

C=1 and γ =0.01

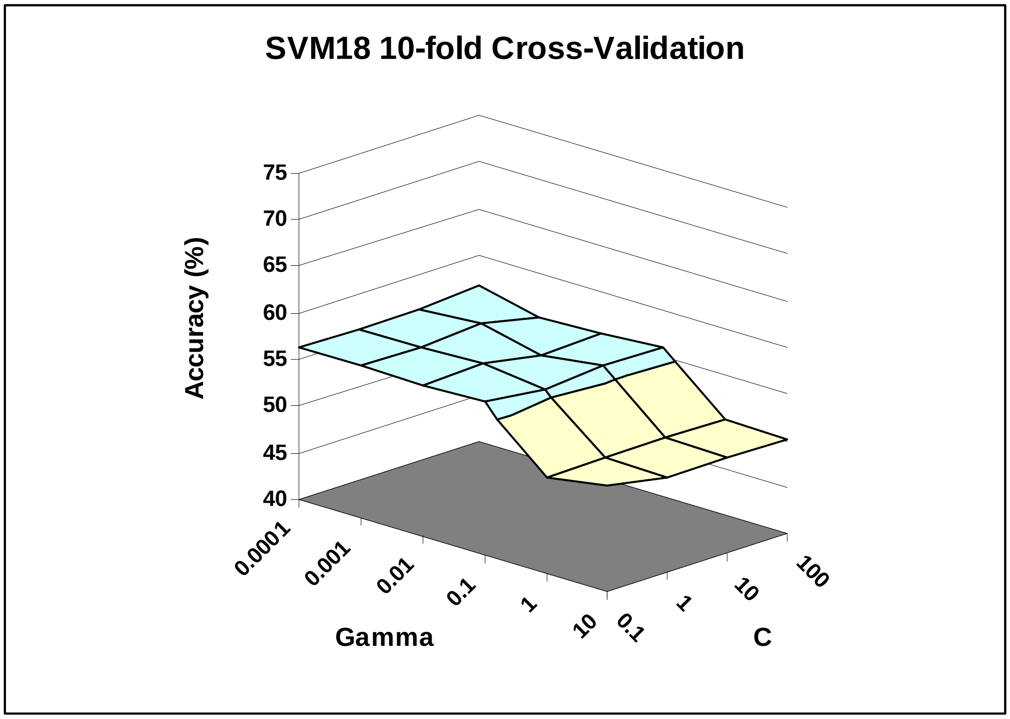

C=10 and γ =0.001

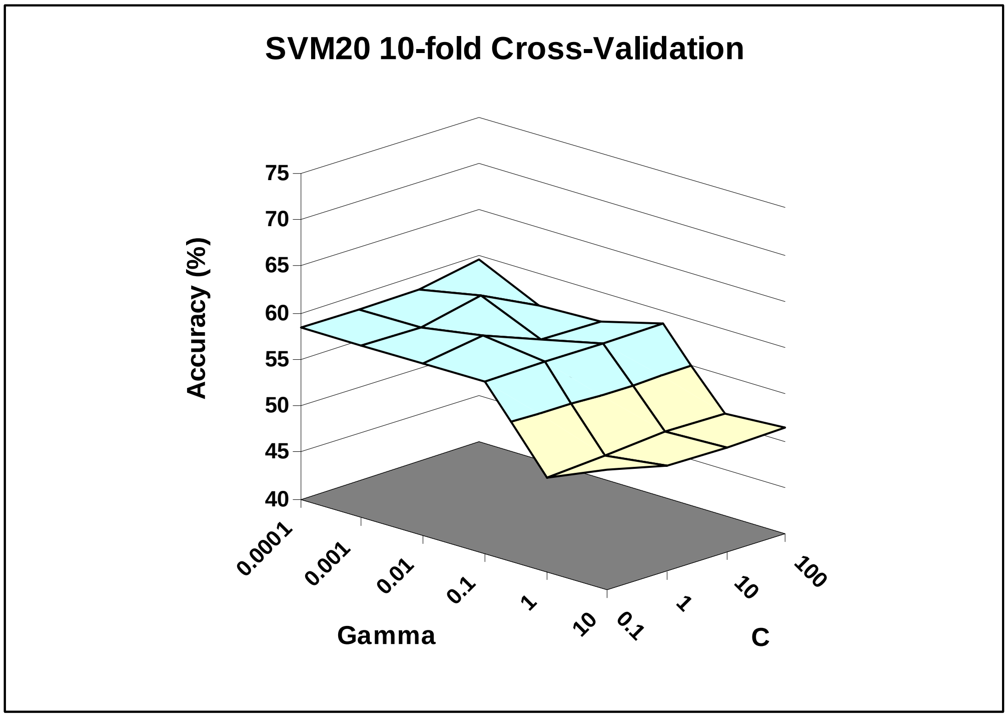

C=10 and γ =0.001

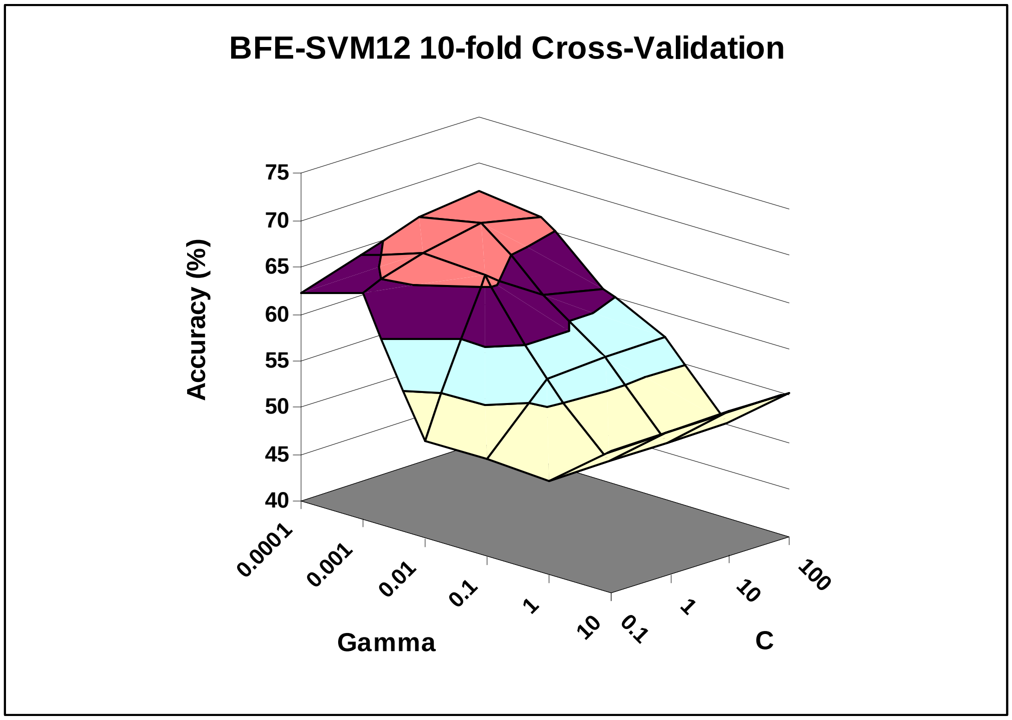

C=100 and γ =0.0001

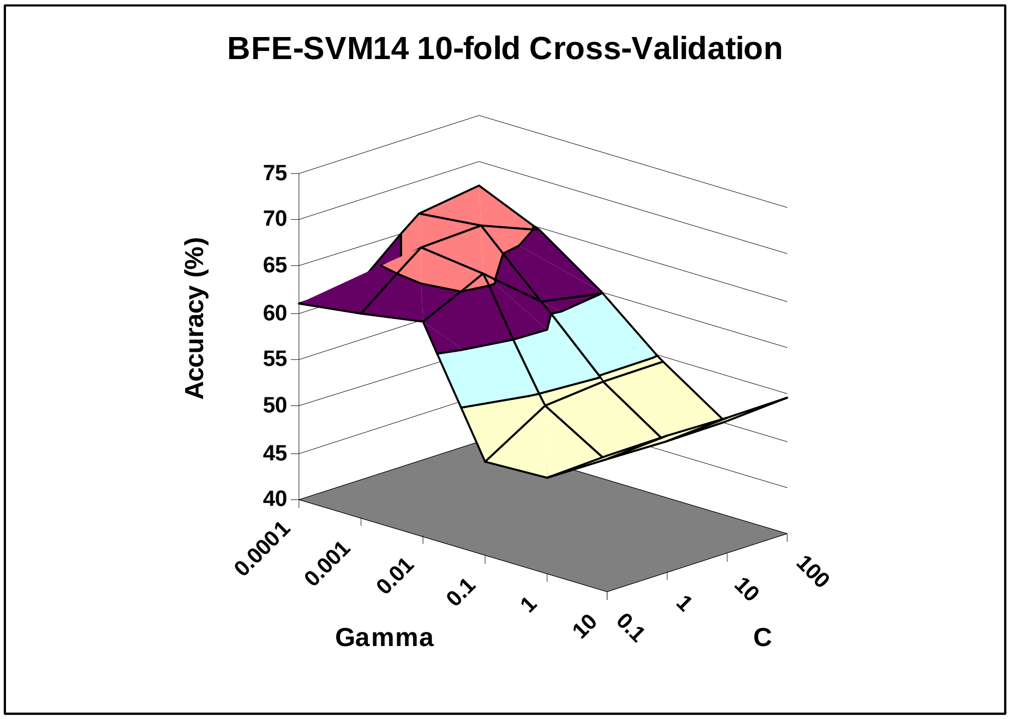

C=100 and γ =0.0001

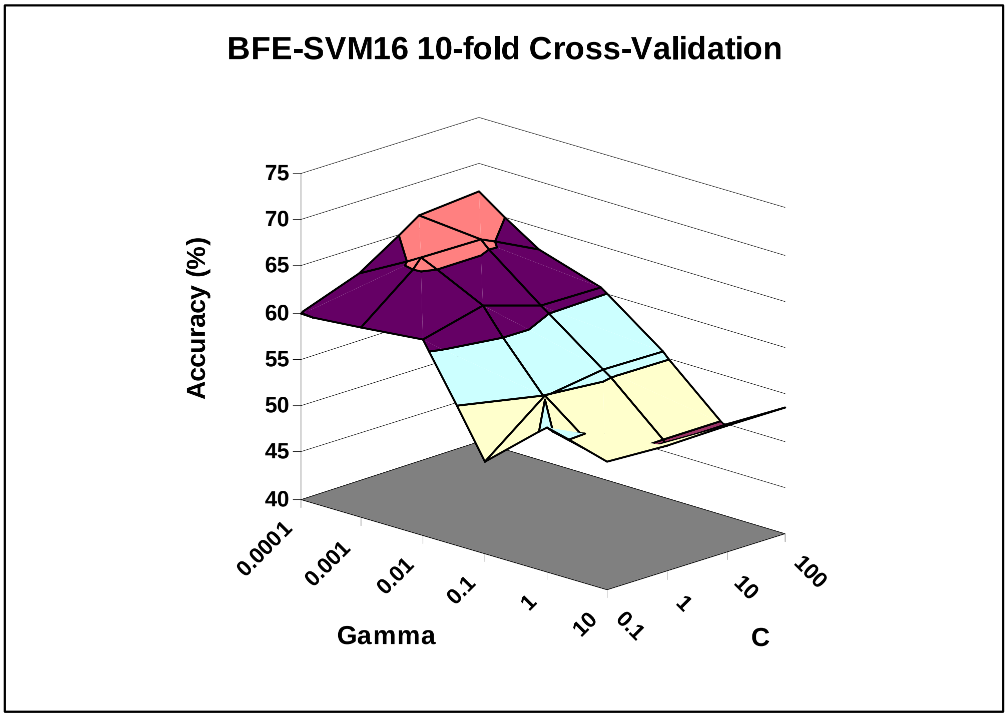

C=100 and γ =0.0001

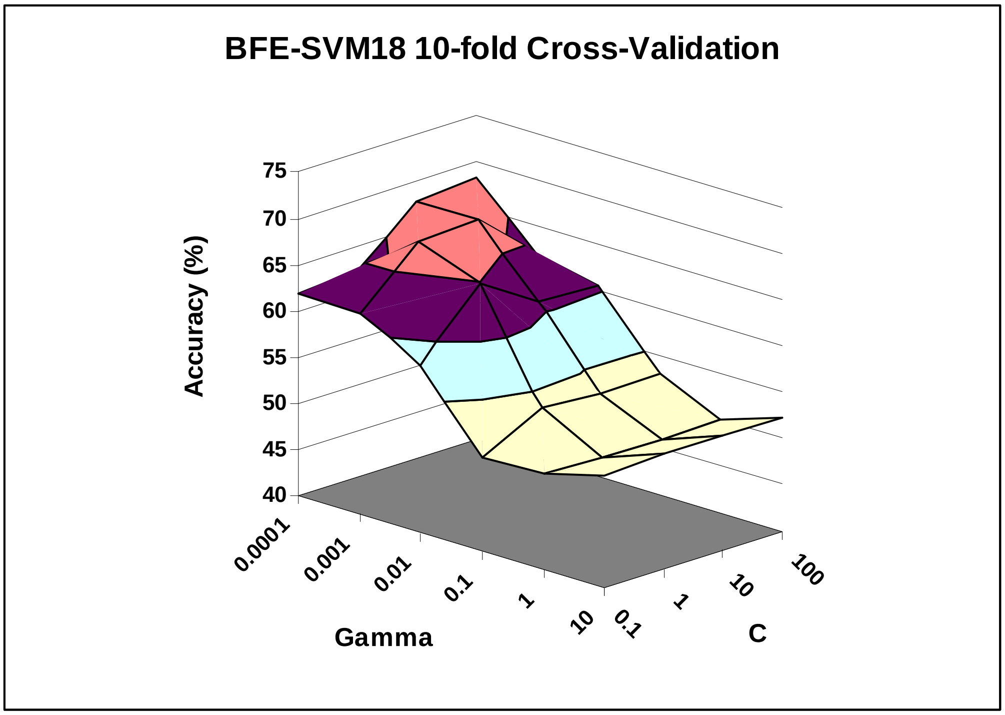

C=100 and γ =0.0001

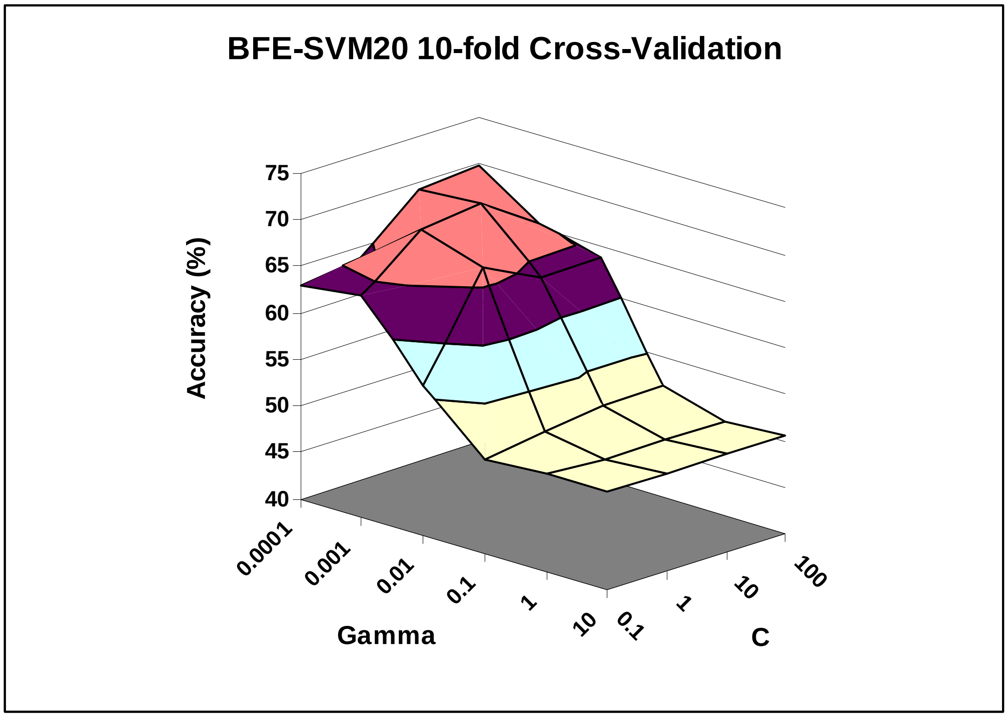

C=100 and γ =0.0001

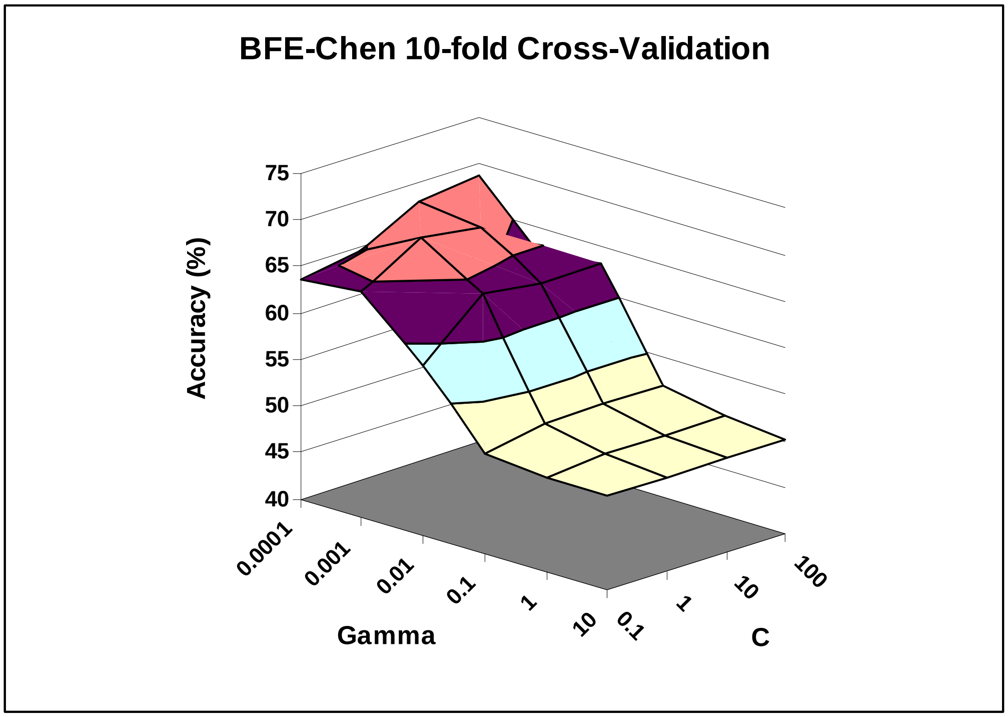

C=100 and γ =0.0001
